# Spatial clustering of suicide mortality and associated community characteristics in Kanagawa prefecture, Japan, 2011–2017

**DOI:** 10.1186/s12888-020-2479-7

**Published:** 2020-02-18

**Authors:** Kazue Yamaoka, Masako Suzuki, Mariko Inoue, Hirono Ishikawa, Toshiro Tango

**Affiliations:** 1 Teikyo University Graduate School of Public Health, 2-11-1, Kaga, Itabashi-ku, Tokyo, 173-8605 Japan; 2 Center for Medical Statistics, Tokyo, Japan

**Keywords:** Suicide, Mortality, Spatial epidemiology, Bayes estimates, Cluster detection test, global test, FleXScan, SaTScan

## Abstract

**Background:**

Suicide mortality is high in Japan and early interventional strategies to solve that problem are needed. An accurate evaluation of the regional status of current suicide mortality would be useful for community interventions. A few studies in Kanagawa prefecture, located next to Tokyo and with the second largest population in Japan, have identified spatial clusters of suicide mortality at regional levels. This study examined spatial clustering and clustering over time of such events using spatial data from regional statistics on suicide deaths.

**Methods:**

Data were obtained from regional statistics (58 regions in Kanagawa prefecture) of the National Vital Statistics of Japan from 2011 to 2017. The standardized mortality ratio (SMR) and Empirical Bayes estimator for the SMR (EBSMR) were used as measures. Spatial clusters were examined by Kulldorff’s circular spatial scan statistic, Tango-Takahashi’s flexible spatial scan statistic and Tango’s test. Linear regression and conditional autoregressive (CAR) models were used not only to adjust for covariates but also to estimate regional effects. The analyses were conducted for each year, inclusive.

**Results:**

Among male suicide deaths, being unemployed (50%) was most frequently related to suicide while among female health problem (50%) were frequent. Spatial clusters with significance detected by FlexScan, SatScan and Tango’s test were few and varied somewhat according to the method used. Spatial clusters were detected in some regions including Kawasaki ward after adjustment by covariates. By the linear regression models, selected variables with significance were different between the sexes. For males, unemployment, family size, and proportion of higher education were detected for several of the years studied while for females, family size and divorce rate were detected over this period. These variables were also observed by the CAR model with 5 covariates. Regional effects were much clearer by considering the spatial parameter for both males and females and especially, Kawasaki ward was detected as a high risk region in many years.

**Conclusion:**

The present results detected some spatial clustering of suicide deaths within certain regions. Factors related to suicide deaths were also indicated. These results would provide important information in policy making for suicide prevention.

## Background

Among the leading causes of death, suicide was recognized as a critical public health issue by the World Health Organization (WHO) in its Comprehensive Mental Health Action Plan [1, 2]. Although substantial reductions in suicide have been detected globally, suicide remains a leading cause of years of life lost in many areas in Japan [3]. Suicide mortality in Japan was among the ten worst in the world and the second highest among Asian countries following South Korea. Therefore, suicide prevention remains an important health issue due to the magnitude of its impact [4]. Factors related to suicide deaths and global action to prevent suicide were summarized by WHO [2]. However, suicide mortality has remarkable heterogeneity in trends across countries and in demographic subgroups such as those based on sex, age, and other factors that warrant further investigation. Early interventions to solve this problem by taking action according to the local situation should be developed.

To provide the accurate regional status of current suicide mortality would serve as a useful tool for evaluations by communities as well as political decision making regarding comprehensive suicide prevention. For these purposes, spatial statistics are widely used to detect geographical disease clusters according to different types of data [5, 6]. To investigate space-time clustering of suicide mortality, many different statistical tests have been proposed to determine whether the suicide risk is relatively high compared with that in surrounding regions or in subsequent time periods [5]. Kulldorff’s spatial scan statistic [7] assuming circular clusters along with Spatial, temporal, or space-time scan (SaTScan) software [8] and Tango and Takahashi’s flexible spatial scan statistic [9, 10] assuming non-circular clusters along with Flexible scan (FlexScan) software [11] have been utilized in a wide variety of epidemiological studies and disease surveillance. Tango’s test [12, 13] has been used for detecting disease clustering in many epidemiological studies. The specific characteristics of the model or test may result in differences in the detection of spatial-clusters; therefore, it is worthwhile to use several methods in such examinations.

As for the application related to spatial epidemiology, there have been several reports of suicide cluster analyses worldwide [14–19]. Namely, a study in New South Wales, Australia, between 2005 and 2013 [14] demonstrated the importance of examining geographical variations in spatial clustering of fatal and non-fatal suicide attempts. A study in Central Brazil during 2000–2010 [15] showed spatial-temporal trends and risk of suicide; a study in Sergipe, Brazil, [16] performed spatial analysis and detected temporal trends in suicide mortality for the period from 2000 to 2015; in Kentucky a spatial epidemiologic investigation.

was performed from 1999 to 2008 [17]; and in Idaho in the US [18] spatial clustering of suicide and associated community characteristics for the period 2010 to 2014 were reported. Also, suicide trends were analyzed in Scotland from 1950 to 2014 in comparison with England & Wales [19] and identified ‘vulnerable’ cohorts for providing opportunities to develop suicide prevention strategies. Despite these studies, thus far, detailed analyses to reveal factors affecting suicide death rates as well as differences between the sexes have not been conducted.

In this study, we focused on suicide deaths in Kanagawa prefecture, located next to Tokyo and that has the second largest population in Japan. Although the number of suicides in Kanagawa prefecture has tended to decrease in recent years, it was still the fourth highest in Japan in 2017. Based on the recognition that many suicides can be prevented by social efforts [2], various background issues related to suicide and causative factors in society have been examined from a multi-faceted perspective. Use of surveys and analyses in line with conditions within a specific community are required [4]. In particular, it is warranted to analyze the actual situation of suicides through the use of vital statistics and to summarize and provide the results of these analyses according to individual municipalities.

There has been no previous study that examined statistically significant clusters of suicide mortality in Kanagawa prefecture, Japan, including analyses to adjust for covariates using regional vital statistics. The detection of clusters may be highly useful in surveillance of suicide, finding factors related to suicide, and making suitable policies to control these factors.

This study used spatial epidemiology focusing on deaths in males and females by suicide in Kanagawa prefecture and examined these data by spatial clustering methods. The aim of this study was to examine spatial clustering and clustering over time using spatial data from regional statistics on male and female suicide deaths in Kanagawa prefecture, Japan by looking at clustering over time after the East Japan Earthquake and Tsunami.

### Current status of suicide deaths in Kanagawa prefecture

**Fig. 1:**
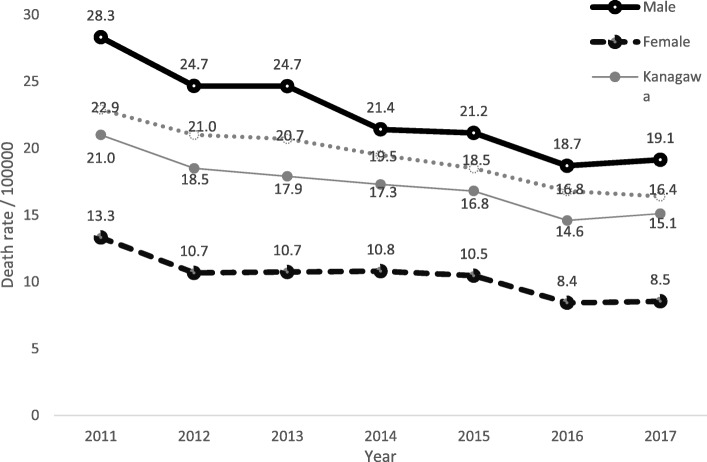
Trend of suicide death rate by sex in Kanagawa prefecture: 2011 to 2017. Note: death rate: per 100000 persons

According to the Kanagawa Prefecture Health Statistics Annual Report [4], among the leading causes of death, suicide was the 7th highest cause of death when all ages were considered. Figure 1 shows the yearly trend of the suicide death rate (per 100000 persons) by sex in Kanagawa prefecture from 2011 to 2017. During this period, suicide deaths had decreased for both males and females. The high male-to-female ratio of suicide mortality (≥2 times) was consistent for a long time regardless of the geographic region.

**Fig. 2:**
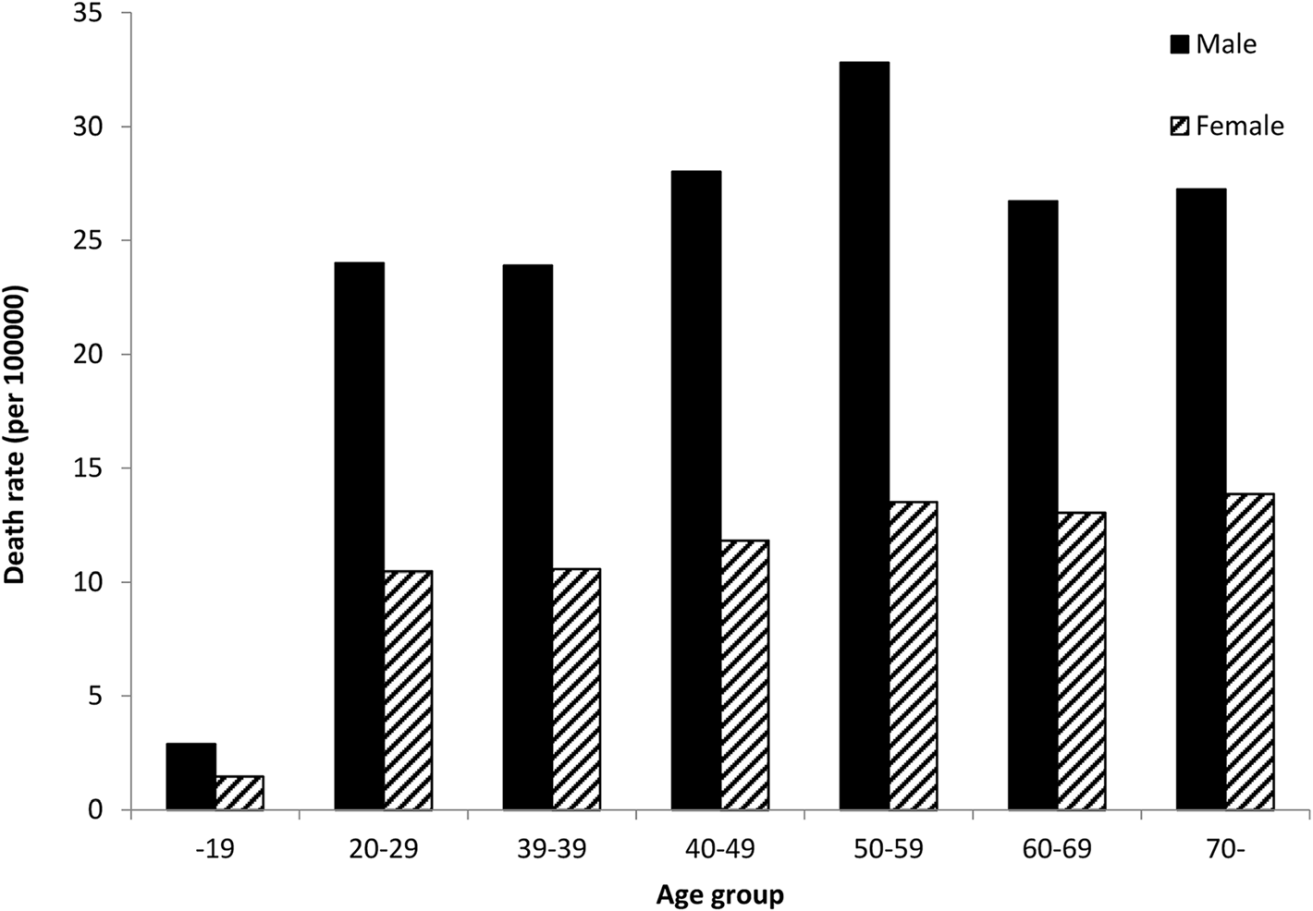
Death rates by suicide according to sex and age group in Kanagawa prefecture

As for death rates by age group, male suicide deaths were highest among those in their fifties, followed by the forties, sixties and seventies while among females, suicide death rates increased with age. (Fig. 2).

**Fig. 3:**
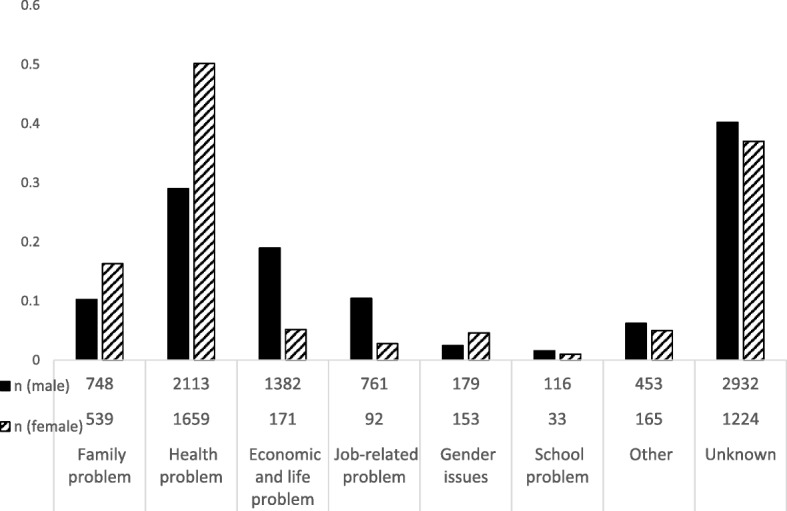
Motivations for suicide during the period from 2011 to 2017

On the other hand, trends by sex for the number of suicides according to specific motivations have not changed over time (see Additional file 1: Material 1). Data from the period 2011 to 2017 indicated that except for “unknown” reasons “Health problems (worries about physical and mental illness)” was the most frequent motivation, followed by “economic and life problems (such as hardship and unemployment)”, “job-related problems”, and “family problems” in that order for males. Among females, the most frequent motivation was “health problems” and the second most frequent motivation was “family problems” (Fig. 3). The high male-to-female ratio for suicide mortality (≥ 2 times) has been consistent for a long time regardless of the geographic regions.

## Methods

The design of this study was a longitudinal follow-up using vital statistics. We used spatial data on regional statistics (58 regions in Kanagawa prefecture) of the National Vital Statistics published by the Cabinet Office, government of Japan, from 2011 to 2017 [20]. These data were downloaded using e-Stat, which is a portal site for Japanese Government Statistics.

### Measures

#### Vital statistics

Age-specific vital data for suicide were used in this study. Age was categorized according to 7 groups (− 19, 20–29, 30–39, 40–49, 50–59, 60–69, 70- years). Using the number of individuals in each age group, indices of standardized mortality were calculated as shown in the Additional file 1: Material 2.

#### Factors

As for covariates, 10 variables identified in the regional data (2015), including the unemployment rate, family size, and population density, were obtained from Japanese National Census data [21]. These data were also downloaded from e-Stat. Table 2 summarizes the characteristics of the variables with the mean value, minimum and maximum values, and corresponding regional number (see Additional file 1: Material 3) shown**.**

### Statistical analyses

We used two indices for standardized mortality rates such as standardized mortality ratio (SMR) ${\hat{\theta}}_i$ and empirical Bayes estimator for SMR using a Poisson-Gamma model (EBSMR) ${\hat{\theta}}_{i, EB}$ (formulas are shown in appendix). For both indices, two standardization criteria such as standardization using each year’s sum from 2011 to 2017 (each year standardization) and standardization by using the total sum of 2011 to 2017 (total year standardization) were used.

We examined the status of suicide risk from spatial epidemiology by using borrow of strength in this study. We mainly examined the following questions. Specifically, (i) are there any spatial clusters for suicide deaths in Kanagawa prefecture, (ii) how large was spatial clustering for estimates of suicide risk in each region, and (iii) what are the factors related to suicide mortality at the cluster level and how would the regional clusters be detected after adjustment for covariates?

In this study, we focused on detecting the spatial clustering of suicide mortality and associated community characteristics in Kanagawa prefecture from 2011 to 2017. Most likely clusters were detected by the following three methods: Kulldorff’s circular spatial scan statistic, Tango-Takahashi’s flexible spatial scan statistic using software FleXScan (which includes Kulldorff’s circular scan statistic) [11], and Tango’s test using software DMS (Disease Mapping System) [22].

In this study, EBSMR was used as the primary measure of the regional health index and Tango-Takahashi’s flexible spatial scan statistic was used as the primary cluster detection test. The results were compared to results of Kulldorff’s circular spatial scan statistic as well as to the results of Tango’s test. Linear regression model using the variable stepwise selection method (inclusion and exclusion criteria: 0.2) for log(EBSMR) as a dependent variable was used to examine the effects of the covariates by each year with the following model.

$$ \begin{aligned} \log\left(\text{EBSMR}_{i}\right) &= \mu + {\beta}_{1} x_{1i} + \cdots + \beta_{10}x_{10i}\\ &\quad + {\epsilon}_{i},\left(x_{1i},..,x_{10i}\right)\\ & :\ \text{covariates}, \epsilon_{i}\sim N\left(0,{\sigma}_{\epsilon}^{2}\right). \end{aligned} $$

Statistical Analysis System (SAS) ver9.4 was used for the analyses. Furthermore, in order to estimate *θ*_*i*_ (SMR) as well as the regression coefficients β_i_ by taking spatial correlation into account, the following conditional autoregressive (CAR) model [23, 24] was considered:

$$ {d}_i\sim \mathrm{Poisson}\left({e}_i{\theta}_i\right) $$

$$ \log {\theta}_i=\mu +{\beta}_1{x}_{1i}+\cdots +{\beta}_2{x}_{2i}+{\beta}_p{x}_{pi}+{\epsilon}_i+{\phi}_i\ \left(p=10\ \mathrm{or}\ 5\right) $$

$$ {\epsilon}_i\sim N\left(0,{\sigma}_{\epsilon}^2\right) $$

$$ {\phi}_i\mid {\phi}_{j\ne i}\sim N\left({\overline{\phi}}_i,\frac{1}{m_i}{\sigma}_{\phi}^2\right) $$

where *ϕ*_*i*_ indicate spatial correlation (also called spatial smoothing parameters), *m*_*i*_ denote the number of regions adjacent to region *i*, ${\overline{\phi}}_i$ denote the average of *ϕ*_*j*_ among regions adjacent to region *i*, and the following three hyperprior distributions were assumed:

$$ \mu \sim \mathrm{improper}\ \mathrm{prior} $$

$$ {\sigma}_{\epsilon}^2\sim \mathrm{Gamma}\left(0.5,0.005\right) $$

$$ 1/{\sigma}_{\phi}^2\sim \mathrm{Gamma}\left(0.5,0.005\right) $$

The CAR models with 10 covariates and 5 covariates (based on the result of the linear regression model) were applied. Furthermore, we examined the regional effects by the CAR model without covariates. All the analyses were conducted by sex. OpenBUGS was used for the analysis and is the open source variant of WinBUGS (Bayesian inference Using Gibbs Sampling) [25].

## Results

Among causes of male suicide deaths age in the 40s (21%) and 50s (18%), being unemployed (50%), living alone (37%), and having family problems (29%) were comparatively frequent causes. For females, age in the 30s, 40s, and 50s (16% for each), being unemployed (79%), living alone (24%), health problems (50%), and family problems (16%) were comparatively frequent causes. (Table 1)

**Table 1:** Demographic characteristics of suicides by sex in Kanagawa prefecture, Japan, 2011–2017

	Male % (*n* = 7292)	Female % (*n* = 3308)
Age groups (years)		
Under 20	14	13
30–39	15	16
40–49	21	16
50–59	18	16
60–69	16	11
Over 70	16	4
Living alone		
Alone	37	24
Unknown	1	0.1
Occupation		
Employee	35	15
Self-employed	8	2
Unemployed	50	79
Unknown	2	1
Suicide at weekend		
Yes	25	29
Suicide method		
Hanging	67	63
Poison	1	3
Firearm	7	3
Jumping from a tall building	7	11
Jumping in front of a train	4	4
Other/unknown	9	11
Causes and motivations^#^		
Family problem	10	16
Health problem	29	50
Economic and life problem	19	5
Job-related problem	10	3
Gender issues	2	5
School problem	2	1
Other	6	5
Unknown	40	37

Note: numbers indicate percentages

#; including multiple answers

Characteristics of covariates in 58 regions in Kanagawa prefecture are shown in Table 2. Unemployment was high in region No. 19 (Kawasaki ward) and family size, population density, proportion of higher education, average income, and proportion of tertiary industries were higher in small towns in rural areas (regions No. 54,55,57, and 58).

**Table 2:** Characteristics of variables in 58 regions in Kanagawa prefecture

Variables	Mean (SD) or median [Q1,Q3]	Minimum value	Region No	Maximum Value	Region No
unemployment (%)	4.2	0.7	2.6	54	5.8
family size	2.3	0.2	1.8	54	2.8
population density (person/km^2^)	6320	[1828, 8705]	44	58	17,540
% of higher education	22.9	7.1	10.2	57	40.6
average income (10,000 yen)	352.9	41.1	277	54	450
death rate (/100,000)	9.2	2.4	5.1	18	16.6
% of the tertiary sector	71.4	5.3	58.2	27	85.7
# of psychiatric hospital	0	[0, 1]	0.0	$	6.0
divorce rate (/1000)	1.7	0.4	0.3	58	2.7
marriage rate (/1000)	4.9	1.4	1.2	58	9.7

$ 31 regions had no psychiatric hospital

**Fig. 4:**
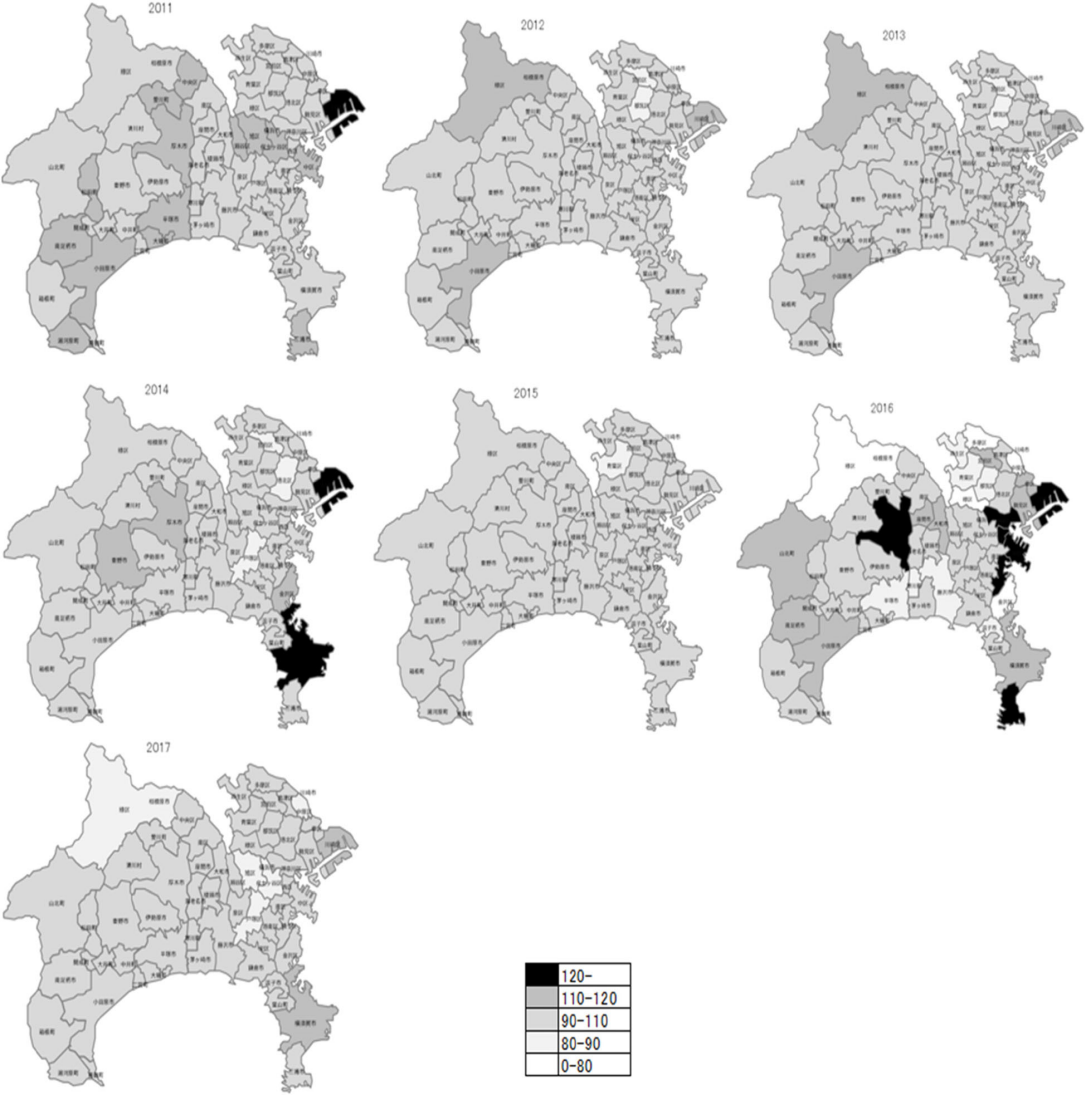
EBSMR for male suicides by year (each year standardization) in Kanagawa prefecture

**Fig. 5:**
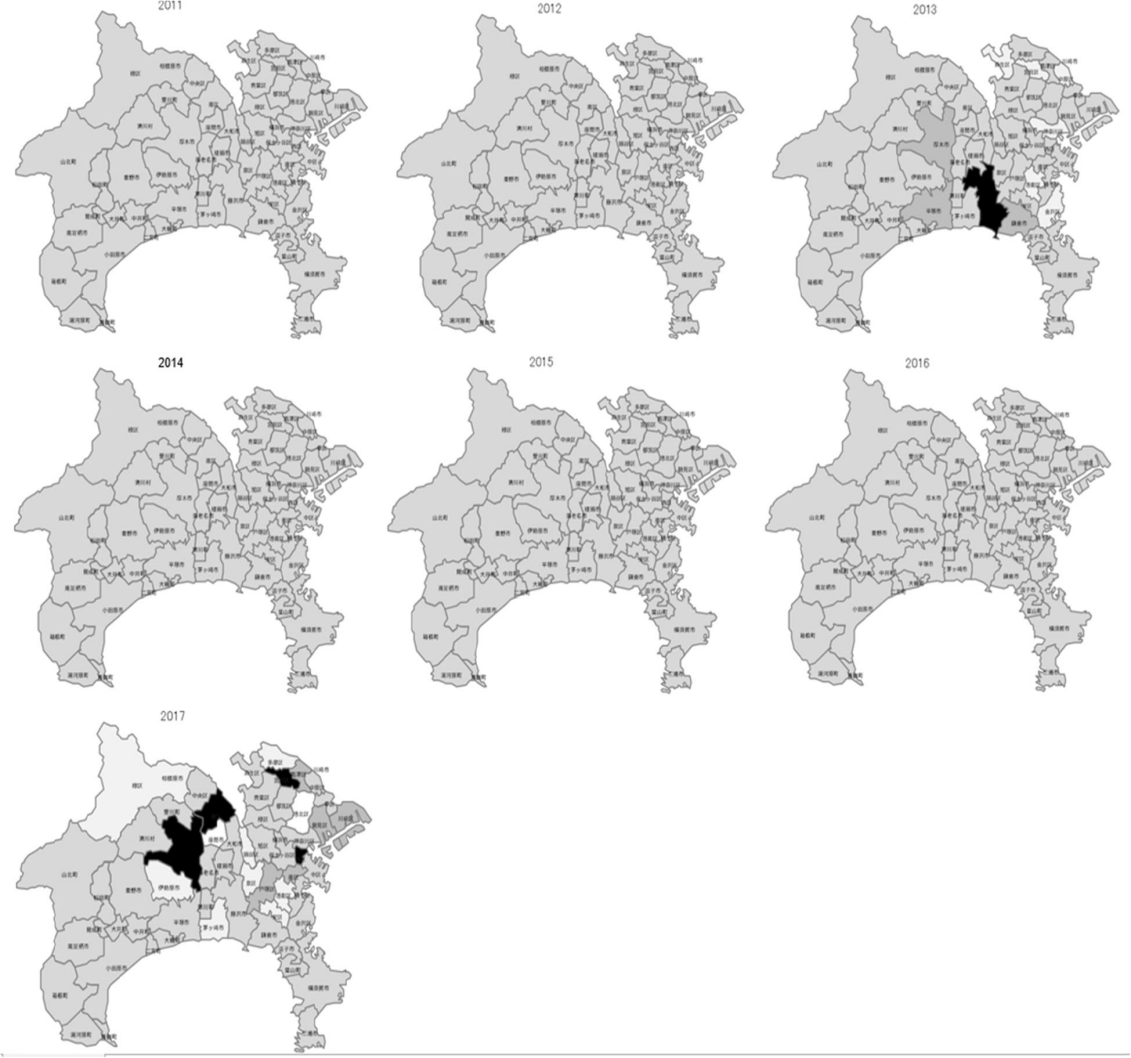
EBSMR for female suicides by year (each-year standardization) in Kanagawa prefecture

In the maps for SMR and EBSMR, the estimates were classified into 5 levels (low: 0–80; somewhat low: 80–90; moderate: 90–110; somewhat high: 110–12; high: 120-). Since the populations in the regions varied (from 2000 to over 200000), the SMR (Additional files 1: Material 4 and 5) varied largely according to region and year that data were collected compared to EBSMR (Figs. 4 and 5). In the case of total year standardization, the EBSMR showed a temporal tendency toward decreases in suicide mortality more clearly compared to data for each-year standardization (Additional file 1: Materials 6 and 7).

The results of several spatial tests as well as regression models are summarized in Table 3 for males and Table 4 for females. Regions detected as having significant spatial clusters by Tango’s test and FlexScan, were few and varied somewhat according to the method used.

**Table 3:** Paramer estiates of covariates: Regression model for log(EBSMR) and CAR models with significantly detescted regions3a (Male)

Variables	2011			2012			2013			2014			2015			2016			2017		
	β	SE	*p*-value	β	SE	p-value	β	SE	p-value	β	SE	p-value	β	SE	p-value	β	SE	p-value	β	SE	p-value
Linear regression model																					
unemployment (%)	0.015	0.008	0.063	–			–			0.039	0.009	<.0001	–			0.051	0.022	0.027	0.017	0.006	0.008
family size	−0.022	0.008	0.006	−0.025	0.007	0.001	−0.025	0.007	0.001	–			−0.008	0.005	0.122	–			–		
poplation density (person/km^2^)	−0.038	0.009	0.000	–			–			–			–			–			–		
% of higher education	−0.035	0.013	0.012	–			–			–			−0.033	0.009	0.000	–			–		
average income (10,000 yen)	0.039	0.012	0.002	–			–			–			0.016	0.009	0.071	–			–		
death rate (/100,000)	–			0.019	0.007	0.005	0.020	0.007	0.004	–						–			–		
% of the tertiary sector	–			−0.028	0.007	0.000	−0.028	0.007	0.000	–			–			–			–		
# of psychiatric hospital	–			–			–			−0.017	0.009	0.066	–			–			–		
divorce rate (/1000)	–			–			–			–			–			–			–		
marriage rate (/1000)	–			–			–			–			–			–			–		
CAR model with 5 covariates	β	lower	upper	β	lower	upper	β	lower	upper	β	lower	upper	β	lower	upper	β	lower	upper	β	lower	upper
unemployment (%)	0.030	− 0.071	0.135	−0.045	−0.150	0.063	−0.042	−0.147	0.066	0.113	−0.007	0.234	−0.002	−0.118	0.117	0.081	−0.087	0.257	0.173	0.002	0.351
family size	−0.106	− 0.200	−0.011	−0.131	−0.230	−0.031	−0.128	−0.226	− 0.026	−0.071	−0.181	0.041	−0.073	−0.182	0.037	−0.034	−0.182	0.132	−0.048	−0.200	0.110
poplation density (person/km^2^)	−0.152	− 0.250	−0.053	−0.078	−0.181	0.026	−0.079	−0.182	0.025	−0.072	−0.186	0.042	−0.005	−0.117	0.106	0.045	−0.115	0.217	0.019	−0.141	0.190
% of higher education	−0.095	− 0.232	0.042	−0.145	−0.289	0.000	−0.145	−0.288	0.000	−0.040	−0.198	0.116	−0.164	−0.322	− 0.008	−0.059	−0.276	0.162	0.079	−0.137	0.302
average income (10,000 yen)	0.087	−0.027	0.201	−0.002	−0.127	0.122	−0.001	−0.126	0.123	0.019	−0.113	0.149	0.015	−0.118	0.150	−0.018	−0.236	0.180	−0.087	−0.283	0.100
Significantly detected regions^$^	[4] [19] [26] [33] [38] [51] [55] [56] [57]			[19] [26] [54] [56] [57]			[19] [26] [54] [56] [57]			[4] [19] [29] [37] [55] [56] [57]			[19] [57]			[4]			[19] [29]		
CAR model with 10 covariates	β	lower	upper	β	lower	upper	β	lower	upper	β	lower	upper	β	lower	upper	β	lower	upper	β	lower	upper
unemployment (%)	0.006	−0.113	0.129	−0.017	−0.146	0.115	−0.016	−0.144	0.116	0.092	−0.044	0.234	0.005	−0.133	0.148	0.045	−0.153	0.261	0.210	−0.019	0.456
family size	0.011	−0.132	0.153	−0.076	−0.229	0.077	0.074	−0.075	0.221	−0.063	−0.224	0.097	0.118	−0.040	0.275	0.020	−0.208	0.258	0.076	−0.181	0.328
poplation density (person/km^2^)	−0.083	−0.224	0.060	−0.042	− 0.166	0.082	−0.074	−0.227	0.080	−0.123	−0.284	0.038	0.018	−0.143	0.180	0.122	−0.068	0.332	0.024	−0.232	0.279
% of higher education	−0.118	−0.236	−0.001	−0.012	− 0.208	0.186	−0.040	−0.165	0.084	−0.074	−0.207	0.059	−0.008	−0.140	0.125	0.123	−0.173	0.439	0.045	−0.163	0.271
average income (10,000 yen)	−0.021	− 0.206	0.165	−0.040	− 0.179	0.098	−0.011	−0.208	0.187	−0.087	−0.297	0.128	−0.110	−0.321	0.104	−0.098	−0.320	0.113	0.238	−0.093	0.604
death rate (/100,000)	0.037	−0.092	0.166	0.151	−0.006	0.305	−0.040	−0.179	0.098	0.040	−0.107	0.186	−0.016	−0.165	0.136	0.233	−0.016	0.468	−0.116	−0.357	0.111
% of the tertiary sector	0.114	−0.035	0.262	−0.111	−0.251	0.027	0.157	0.000	0.311	−0.033	−0.209	0.138	0.124	−0.047	0.292	−0.101	−0.332	0.115	0.076	−0.176	0.329
# of psychiatric hospital	−0.024	−0.155	0.108	0.026	−0.034	0.085	−0.110	−0.250	0.029	0.015	−0.134	0.162	0.023	−0.125	0.172	0.028	−0.063	0.121	−0.116	−0.380	0.123
divorce rate (/1000)	0.027	−0.029	0.082	0.010	−0.118	0.137	0.026	−0.034	0.085	−0.063	−0.129	0.002	0.031	−0.034	0.094	0.124	−0.061	0.303	−0.020	−0.122	0.085
marriage rate (/1000)	0.053	−0.064	0.170	0.075	−0.073	0.223	0.007	−0.121	0.134	−0.030	−0.167	0.106	0.024	−0.114	0.161	0.028	−0.204	0.259	0.084	−0.115	0.288
Significantly detected regions^$^	[4] [19] [33] [56]			[19] [33] [57]			[19] [33] [36] [52] [55] [57]			[19] [29] [56]			[19] [33] [36] [55] [56] [57]			[4] [36]			[19] [29]		
Tango’s Index Significantly detected regions^$^	–			–			–			–			–			–			[24] [28] [38]		
FlexScan Significantly detected regions^$^	–			–			–			–			–			[1] [2] [3] [4] [7]			–		
SaTScan Significantly detected regions^$^	–			–			–			[19]			–			[4]			[19] [29]		

β: parameter estimateby multiple regression model with stepwise variable selection method (inclusion and exclustion criteria: 20%)

SE: standard error

-: Variable not included the final model

All variables were centroided (mean 0, variance 1)

CAR model by OpenBUGS (iterations = 100000, Chain = 2, Thining = 10, Sample = 20000)

$: Region number (see Additional file 1: Material 3)

**Table 4:** Paramer estiates of covariates: Regression model for log(EBSMR) and CAR models with significantly detescted regions3a (Female)

Variables	2011			2012			2013			2014			2015			2016			2017		
	β	SE	p-value	β	SE	p-value	β	SE	p-value	β	SE	p-value	β	SE	*p*-value	β	SE	p-value	β	SE	p-value
Linear regression model																					
unemployment (%)	–			–			–			–			–			–			–		
family size	–			–			−0.025	0.012	0.039	–			−0.007	0.004	0.080	−0.011	0.004	0.006	–		
poplation density (person/km^2^)	–			–			−0.039	0.012	0.002	–			–			–			–		
% of higher education	–			–			–			–			−0.012	0.004	0.004	–			–		
average income (10,000 yen)	–			–			–			–			–			–			–		
death rate (/100,000)	–			–			–			−0.593	0.163	0.001	–			–			–		
% of the tertiary sector	–			–			–			0.420	0.164	0.013	–			−0.004	0.003	0.147	–		
# of psychiatric hospital	–			–			0.017	0.010	0.087	–			–			–			–		
divorce rate (/1000)	0.007	0.004	0.099	0.007	0.002	0.007	–			0.477	0.164	0.005	–			0.004	0.002	0.096	–		
marriage rate (/1000)	–			–			–			–			–			−0.011	0.003	0.003	–		
CAR model with 5 covariates	β	lower	upper	β	lower	upper	β	lower	upper	β	lower	upper	β	lower	upper	β	lower	upper	β	lower	upper
family size	−0.085	−0.228	0.058	0.042	−0.109	0.195	−0.151	−0.311	0.007	−0.033	−0.186	0.122	0.047	−0.118	0.217	−0.144	−0.317	0.031	−0.101	−0.296	0.091
poplation density	−0.025	−0.160	0.111	−0.064	−0.214	0.085	−0.206	−0.360	−0.050	−0.065	−0.214	0.084	−0.099	−0.252	0.058	−0.107	−0.274	0.060	0.007	−0.174	0.189
% of higher education	0.030	−0.112	0.173	0.020	−0.127	0.167	−0.003	−0.159	0.153	−0.015	−0.164	0.134	0.021	−0.137	0.179	−0.027	−0.202	0.147	−0.007	−0.202	0.186
% of the tertiary sector	−0.019	−0.169	0.131	0.175	0.018	0.333	−0.022	−0.180	0.138	0.089	−0.068	0.248	−0.066	−0.286	0.151	0.026	−0.152	0.205	−0.029	−0.231	0.175
divorce rate (/1000)	0.115	−0.032	0.262	0.305	0.147	0.461	0.027	−0.136	0.188	0.116	−0.044	0.275	−0.097	−0.283	0.088	0.127	−0.053	0.308	0.025	−0.178	0.222
Significantly detected regions^$^	[4]			[4] [14] [19] [45] [51]			[19] [38]			–			[27]			[4] [19]			[19] [29]		
CAR model with 10 covariates	β	lower	upper	β	lower	upper	β	lower	upper	βSignificantly detected regions	lower	upper	β	lower	upper	β	lower	upper	β	lower	upper
unemployment (%)	0.006	−0.113	0.129	−0.017	−0.146	0.115	−0.016	− 0.144	0.116	0.092	−0.044	0.234	0.005	−0.133	0.148	0.045	−0.153	0.261	0.210	−0.019	0.456
family size	0.011	−0.132	0.153	−0.076	−0.229	0.077	0.074	−0.075	0.221	−0.063	−0.224	0.097	0.118	−0.040	0.275	0.020	−0.208	0.258	0.076	−0.181	0.328
poplation density (person/km^2^)	−0.083	−0.224	0.060	−0.042	−0.166	0.082	−0.074	−0.227	0.080	−0.123	−0.284	0.038	0.018	−0.143	0.180	0.122	−0.068	0.332	0.024	−0.232	0.279
% of higher education	−0.118	−0.236	−0.001	−0.012	−0.208	0.186	−0.040	−0.165	0.084	−0.074	−0.207	0.059	−0.008	−0.140	0.125	0.123	−0.173	0.439	0.045	−0.163	0.271
average income (10,000 yen)	−0.021	−0.206	0.165	−0.040	−0.179	0.098	−0.011	−0.208	0.187	−0.087	−0.297	0.128	−0.110	−0.321	0.104	−0.098	−0.320	0.113	0.238	−0.093	0.604
death rate (/100,000)	0.037	−0.092	0.166	0.151	−0.006	0.305	−0.040	−0.179	0.098	0.040	−0.107	0.186	−0.016	−0.165	0.136	0.233	−0.016	0.468	−0.116	−0.357	0.111
% of the tertiary sector	0.114	−0.035	0.262	−0.111	−0.251	0.027	0.157	0.000	0.311	−0.033	−0.209	0.138	0.124	−0.047	0.292	−0.101	−0.332	0.115	0.076	−0.176	0.329
# of psychiatric hospital	−0.024	−0.155	0.108	0.026	−0.034	0.085	−0.110	−0.250	0.029	0.015	−0.134	0.162	0.023	−0.125	0.172	0.028	−0.063	0.121	−0.116	−0.380	0.123
divorce rate (/1000)	0.027	−0.029	0.082	0.010	−0.118	0.137	0.026	−0.034	0.085	−0.063	−0.129	0.002	0.031	−0.034	0.094	0.124	−0.061	0.303	−0.020	−0.122	0.085
marriage rate (/1000)	0.053	−0.064	0.170	0.075	−0.073	0.223	0.007	−0.121	0.134	−0.030	−0.167	0.106	0.024	−0.114	0.161	0.028	−0.204	0.259	0.084	−0.115	0.288
Significantly detected regions^$^	[4] [19] [33] [56]			[19] [33] [57]			[19] [33] [36] [52] [55] [57]			[19] [29] [56]			[19] [33] [36] [55] [56] [57]			[4] [36]			[19] [29]		
Tango’s Index Significantly detected regions^$^	**–**			**–**			**–**			**–**			**–**			**–**			[24] [28] [38]		
FlexScan Significantly detected regions^$^	**–**			**–**			**–**			**–**			**–**			**–**			**–**		
SaTScan Significantly detected regions^$^	**–**			**–**			**–**			**–**			**–**			**–**			**–**		

β: parameter estimateby multiple regression model with stepwise variable selection method (inclusion and exclustion criteria: 20%)

SE: standard error

-: Variable not included the final model

All variables were centroided (mean 0, variance 1)

CAR model by OpenBUGS (iterations = 100000, Chain = 2, Thining = 10, Sample = 20000)

$: Region number (see Additional file 1: Material 3)

**Fig. 6:**
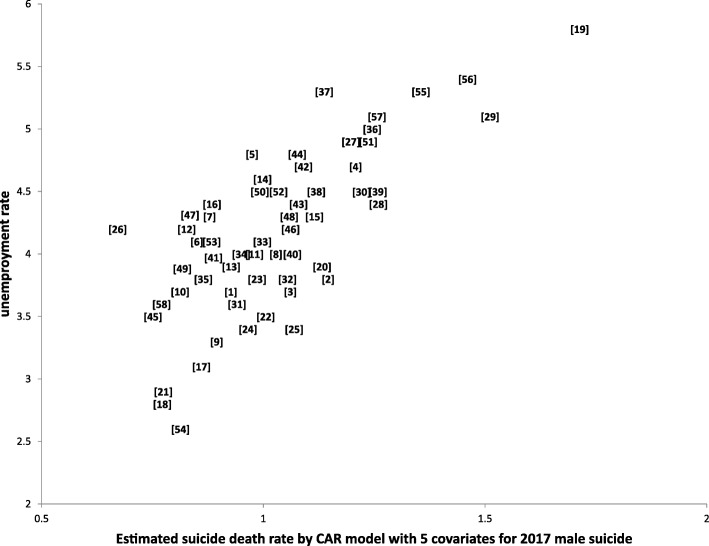
Scatter plot of estimated suicide death rate by CAR model and unemployment rate for males. *Note*: numbers denotes region code. CAR model was conducted with 5 covariates

Especially among females, by the 3 methods, only in 2017 were such regions detected. By the CAR model only including the smoothing parameter (without covariates) showed no significant regional cluster for both males and females (results are not shown). However, with CAR models with covariates, regional tendencies became much clearer by considering the spatial parameter for both males and females and more regions were detected compared to the model that did not include a spatial parameter. Specifically Kawasaki ward was detected as being a significantly higher suicide region for both males and females. Figure 6 shows a scatter plot of the estimated suicide death rates by the CAR model with 5 covariates and the unemployment rate for males. The numbers in the scatter plot denote the region number. This figure shows that Kawasaki ward was the highest both for suicide rate and unemployment rate.

As for the factors related to suicide death rates, by the linear regression model, the selected variables were different between the sexes. For males, unemployment (in 2014, 2016, 2017), family size (in 2011, 2012, 2013), and the proportion of higher education (in 2011, 2015) were detected to be significant in two or more years. However, for females, family size (in 2013, 2016) and the divorce rate (in 2012, 2014) were detected to be significant in two or more years. As to the CAR model with 10 covariates, no significant variables were detected except for the proportion of higher education in 2011 for both males and females and the proportion of tertiary industries in 2013 for females. The results by the CAR model with 5 covariates for males showed the significant protective effects of family size (in 2011, 2012, 2013), population density (in 2011), and proportion of higher education in 2015). In the case of females, a protective effect of population density in 2013 was observed and the proportion of tertiary industries (in 2012) and the divorce rate (in 2012) were related to greater suicide deaths.

## Discussion

Using vital statistics and census data from National Vital Statistics published by the Cabinet Office, government of Japan, from 2011 to 2017 [20], this study evaluated suicide mortality among males and females in Kanagawa prefecture by spatial epidemiology. Results of the analyses showed evidence of hotspots of suicide mortality across regions in Kanagawa prefecture and revealed related factors. To our knowledge this is the first study to investigate spatial and temporal patterns of suicide mortality by considering related factors at the regional level in Japan. During this period, the suicide rates in Kanagawa prefecture for each year were lower than those of Japan as a whole (Fig. 1). Although suicide mortality has been gradually decreasing in Kanagawa prefecture since 2011, some regions within the prefecture were still comparatively high-risk regions. The results of this study can be expected to bring a better understanding of where to target resources and preventive efforts at the regional level to reduce the burden of suicide in high-risk regions.

Many studies have used spatial epidemiological technics especially for infectious diseases. These studies employed spatial epidemiology, which was used to describe and analyze geographic variations in disease with respect to demographic, environmental, behavioral, socioeconomic, genetic, and infectious risk factors [26, 27]. These methods are useful not only for studies of infectious diseases but also for studies of non-infectious diseases or events like suicide. Worldwide there have been several reports of suicide cluster analyses [14–19]. We performed spatial cluster analyses using several methods as well as examined related factors using the CAR model including spatial smoothing. Thus far, detailed analyses such as ours have not been conducted as mentioned in the Background section. That these analyses revealed that factors affecting suicide death rates differed between the sexes and among the years studied might be useful in formulating community countermeasures.

According to the report by WHO [2], in high-income countries three times as many males die by suicide as females. In Kanagawa prefecture, the male-to-female ratio for suicide indicated that twice as many males as females committed suicide (Table 1, Fig. 1), with suicide deaths more frequent in persons in their 40s and 50s. In our study, Kawasaki ward (region number 19) was revealed to be a hotspot irrespective of differences in the models employed. The EMSMRs in Kawasaki ward varied from 110.8 to 131.2 for males and 101.6 to 111.1 for females during 2011 to 2017; these values were the largest among regions within Kanagawa prefecture. Furthermore, the unemployment rate was the largest in Kawasaki ward (5.8%). (see Fig. 6) Kawasaki ward is an urban industrial zone and is located in a coastal area (Kawasaki Port), the Keihin Industrial Area, where information service industries, large oil complexes, and steel works are concentrated as well as many large factories.

The main industry is manufacturing, and daily employment is still present. Furthermore, Kawasaki ward has fewer medical clinics than Kanagawa prefecture as a whole. Regional medical resource data from the Japan Medical Association Regional Medical Information System showed that there are fewer clinics per 100000 people in Kawasaki ward compared to Kanagawa prefecture (Kawasaki ward vs. Kanagawa prefecture: 52.83 vs. 68.14), with few psychiatric clinics per 100000 people (5.37 vs. 6.52). The scarcity of clinics could hamper early detection/continuation of treatment of depression, alcohol dependence, etc. In fact our study results suggested that difficulties in access to clinics might be one reason for the high suicide mortality in Kawasaki ward.

Results of a quasi-experimental study conducted in Japan [28] to examine the effectiveness of the Multimodal Community Intervention Program to Prevent Suicide suggested the effectiveness of interventions for males and the elderly in rural areas to prevent suicide. Suicide prevention has been considered to be an important priority in policy making [29]. The incredibly rapid change in society throughout Japan may have resulted in difficulties in forming social networks such as neighborhood relationships because individuals move into an area primarily because of the workplace rather than to create a permanent household. Also, the welfare utilization rate is high, the foreign population is large, and disparities in the social environments are large even within the ward.

Many suicide attempters belong to highly vulnerable marginalized groups. Especially, the young and elderly are considered most susceptible to suicidal ideation and self-harm. However, in this study, the 40th and 50th were recognized as vulnerable, and men in that age group can be considered to be marginalized with regard to suicide possibly because of working conditions and social roles. Men in midlife tend to devote themselves to work and family and lack outside social connections. One study based on the National death certification, issued by a registrar of vital statistics for official register of deaths, suggested that middle-aged Japanese men among management workers rather than clerks and blue-collar workers tend to commit suicide [30]. These findings imply the necessity of introducing or improving workplace mental health policies and services. The Japanese government set the General Principles of Suicide Prevention Policy in 2007, and that document suggested the use of gatekeepers, volunteers who assist the vulnerable by offering services such as helplines in society for suicide prevention [31]. Further research is necessary to evaluate the impact of policies for middle-aged men to prevent suicide. Policies including those on statistical research and provision of information on suicide prevention measures have been cited as new developments as has analysis of the actual situation of suicide in specific areas.

Suicide prevention addresses a wide range of social factors. Furthermore, it is important to note that since suicide is affected by sociocultural factors, effective interventions in a certain population may not work in another population. Therefore, since preparing profiles of actual local suicides should be important, our study can provide useful information. In addition, our finding that unemployment was frequently associated with suicide deaths was similar to the finding that socioeconomic deprivation has been associated with high-risk suicide clusters detected in the above-mentioned studies.

### Strengths, limitations, and future research

One strength of this study is that it used novel spatial clustering techniques from the viewpoint of spatial epidemiology to provide ecological information on suicide risk. Thus our results have the potential to guide interventions in high-risk regions. In addition, these methods are easily applicable to other areas. Spatial analyses can be performed using suicide census data at the regional level and it would allow the visualization of high-risk suicide clusters.

As for the limitations of our study, firstly our data on socioeconomic and environmental factors were limited to only one point in the census year (2015). However, a further investigation determining the factors associated with clusters of individuals at high risk of suicide using various sociodemographic and environmental regional characteristics by the CAR model may reveal useful information. Secondly, our analyses were performed using data from groups. The modifiable areal unit problem (MAUP) is a source of statistical bias that can significantly impact the results of statistical hypothesis tests. Although we cannot deny the possibility of bias, considering that the reasons for suicide death (Table 1) and the result of the CAR models seemed to be somewhat similar to each other, the effects of the MAUP may not be largely affected. If individual personal data were available, a detailed analysis would be possible. This is another limitation of our study. Although our analysis only clarified high-risk regions in Kanagawa prefecture, the analytical process may be applied to other regions not only in Japan but also in other countries.

## Conclusion

Statistically significant spatial clusters of populations at high risk of suicide were detected at the regional level. The present results using spatial clustering of suicide data in Kanagawa Prefecture detected some regions within the prefecture that were at high risk. These results would provide important information for policy making on suicide prevention.

## Supplementary information

**Additional file 1.** Supplementary material 1. The proportion of suicides according to specific motivations during the 2011 to 2017 by year. Supplementary material 2. Indices for standardized mortality. Supplementary material 3. Regional code of Kanagawa prefecture. Supplementary material 4. SMR for male suicide by year (each year standardization) in Kanagawa prefecture. Supplementary material 5. SMR for female suicide by year (each year standardization) in Kanagawa prefecture. Supplementary material 6. EBSMR for male suicide by year (total year standardization) in Kanagawa prefecture. Supplementary material 7. EBSMR for female suicide by year (total year standardization) in Kanagawa prefecture.

## Data Availability

All data were applicable from the following hyperlinks to publicly archived datasets analyzed:

## Abbreviations

CAR: Conditional autoregressive
DMS: Disease Mapping System
EBSMR: Empirical Bayes estimator for the SMR
FlexScan: Flexible scan
MAUP: Modifiable areal unit problem
OpenBUGS: Open source version of Bayesian inference Using Gibbs Sampling
SAS: Statistical Analysis System
SaTScan: Spatial, temporal, or space-time scan
SMR: Standardized mortality ratio
WHO: World Health Organization
